# Dalbavancin to Treat Infected Massive Endoprostheses: A Case Report and Cost Comparison Analysis

**DOI:** 10.7150/jbji.37980

**Published:** 2019-10-15

**Authors:** Tariq Azamgarhi, James Donaldson, Ashik Shah, Simon Warren

**Affiliations:** Royal National Orthopaedic Hospital

**Keywords:** Periprosthetic joint infection, Lipoglycopeptide, Coagulase negative staphylococcus epidermidis, Osteomyelitis

## Abstract

We report a case of an infected massive endoprosthetic replacement treated successfully with 2 stage surgery and off-label dalbavancin. Dalbavancin was used due to a limited number of antimicrobial options that could be administered safely in an outpatient setting and to avoid the need for daily dosing.

## Background

Endoprosthetic replacement (EPR) is often used in complex cases of revision arthroplasty where bone loss and instability are a problem. Deep infection following these procedures is a devastating complication, often requiring further surgery and prolonged antimicrobial treatment 1. The option of Outpatient Parenteral Antimicrobial Therapy (OPAT) improves the quality of life of patients, reduces the risk of hospital-acquired infections and healthcare costs 2. Common causative organisms include Coagulase negative staphylococci (CNS) that have the potential to develop resistance to multiple antimicrobials 3. Antimicrobial intolerances can further limit the choice of antimicrobials that can be administered safely in an outpatient setting.

Dalbavancin is a second generation semisynthetic lipoglycopeptide with activity against CNS. Its half-life of 14 days (range 6.1 to 18.4) is significantly longer than other antimicrobials, and is currently approved for single dose intravenous treatment for acute bacterial skin and skin structure infections (ABSSSI) 4.

Case reports describing its use to treat bone and joint infections are rare, and to our knowledge, none of these describe use for infected EPR or the dosing regimen of dalbavancin used 5,6. We report a case of late-onset infected EPR caused by a multi-resistant *Staphylococcus epidermidis* treated successfully with dalbavancin.

## Case report

This case involves a 76-year-old Caucasian female with a history of bilateral total knee, and total hip replacements (THR) for osteoarthritis 20 years prior. She had 2 revisions on her left hip, the first within 5 years of surgery and the second 19 years ago with no further problems on that side. She had 5 previous revisions on the right side; 2 initial revisions due to wound infections, one following a fall, a further revision due to an external rotation defect and the most recent being a 2 stage revision due to infection with CNS. This revision involved conversion to a custom-made proximal femoral replacement 19 months prior to presentation.

She presented to outpatients at the Royal National Orthopaedic Hospital (RNOH) with a 3-month history of right hip pain that was unresponsive to analgesia. On examination she was apyrexial and her right hip was cool, non-inflamed and well healed. Blood tests revealed a slightly raised C-reactive protein (CRP) of 11 mg/L, normal erythrocyte sedimentation rate (ESR) of 6mm/hr and neutrophil count of 7.7 × 10^9^ cells/L. A recent X-ray of the hip showed extensive radiolucency at the cement-bone interface, most marked posteriorly, which was highly suggestive of loosening of the femoral component.

The patient underwent an ultrasound-guided aspiration of the hip and a single joint fluid sample was sent to the Microbiology Department at the Royal Free Hospital, London, UK. Three days later, *Staphylococcus epidermidis* resistant to rifampicin was cultured. A two-stage revision strategy was recommended by the bone infection multi-disciplinary team (MDT).

At the 1st stage, a soft tissue debridement was performed and the stem and cup were removed together with cement. Eight deep tissue samples were taken and a loosely cemented (impregnated with Vancomycin and Gentamicin 1g per 40g of each) silver coated modular subtotal proximal femoral replacement, METS (Stanmore Implants Worldwide, Stanmore, UK) was fitted and the wound closed with drainage. On days 1 to 5 after surgery the patient recieved broad spectrum antibiotic therapy with intravenous vancomycin (1g 12 hourly adjusted according to levels), ceftriaxone (2g once daily), and two doses of amikacin (15 mg/kg once daily). Multi-resistant *Staphylococcus epidermidis* was grown in all 8 samples confirming a diagnosis of PJI based on published criteria 1. Antimicrobial susceptibilities were tested using the BD Phoenix^TM^ system and were sensitive to vancomycin, linezolid, daptomycin and resistant to oxacillin, teicoplanin, gentamicin, rifampicin, ciprofloxacin, clindamycin, tetracycline, and trimethoprim.

Intolerances during previous antimicrobial treatment included a rash due to linezolid and a significantly raised creatine phosphokinase (CPK) >3000 (reference range 25 - 200 units/L) with dyspnoea whilst on daptomycin. To avoid a prolonged inpatient stay on intravenous vancomycin, we considered dalbavancin. Susceptibility was determined by Liofilchem® MIC Test Strips (manufactured by Liofilchem) (MIC≤0.047) in accordance with the recommendations from the European Committee on Antimicrobial Susceptibility Testing (EUCAST). Approval was obtained from the local Drugs and Therapeutics Committee for off-label use.

The first dose of 1500mg was administered on day 6 following surgery. By day 9 the wound was dry, she was mobilising well and bloods showed a neutrophil count of 7.3 × 10^9^ cells/L, CRP of 16 mg/L and a serum creatinine of 38 umol/L (reference range 44 - 80 umol/L). She was discharged on day 9 and the second dose of 1500mg was administered at her local hospital 7 days later. Both doses were well tolerated and with no adverse events. Routine blood tests, including full blood count, renal function and liver function tests were monitored weekly for 8 weeks and remained normal.

Six weeks post operatively the clinical response was satisfactory with complete healing of the wound. The CRP remained slightly raised at 16 mg/L and so a repeat biopsy was requested. This was performed at 4 months following first stage revision and revealed a normal synovial white cell count with one of one tissue samples with growth of *Micrococcus luteus* which was considered a contaminant.

**Figure 1:**
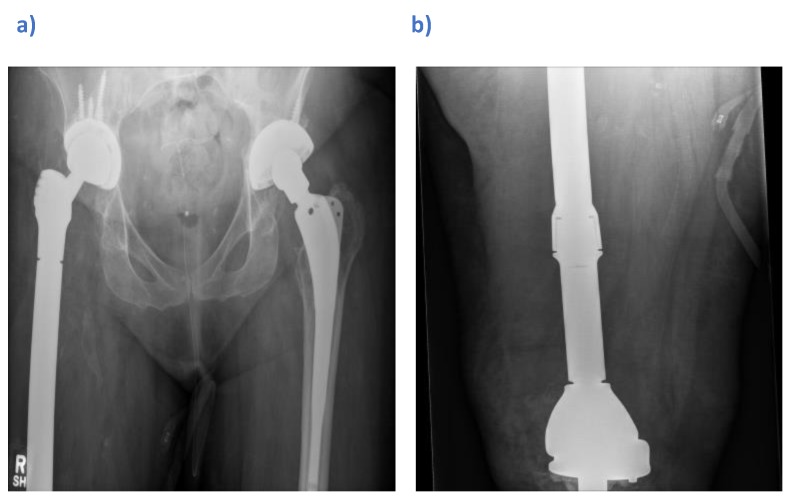
Post-operative radiographs showing a custom-made right total femoral replacement (Stanmore Implants Worldwide, Stryker) a) Anteroposterior view of the pelvis b) Lateral view of the femur

At the second stage, a definitive custom-made total femoral replacement (Stanmore Implants Worldwide, Stryker) was used to include a revision of the knee due to the close proximity and limited remaining bone stock. This was re-implanted 6 months following the first stage revision, with vancomycin impregnated bone graft (Osteomycin) and broad spectrum antimicrobials started post-operatively (Fig 1a and b). Five deep intraoperative tissue samples remained negative after 14 days. Antimicrobials were stopped after 5 days and by day 11 post-operatively, the patient's wound had healed and she was discharged.

At 16 months post re-implantation the patient was reviewed as an outpatient and reported no pain in her hip, and the site was well healed with no clinical signs of infection.

## Discussion

This case involves an elderly female with an infected proximal femoral replacement due to multi-drug resistant *Staphylococcus epidermidis* and multiple antibiotic intolerances successfully treated as an outpatient with off-label dalbavancin. Vancomycin was not preferred as it would require a prolonged inpatient stay for daily dosing and therapeutic drug monitoring. Tedizolid was considered but there were concerns of potential cross-reactivity with linezolid.

Currently, the only licensed dosing regimens for dalbavancin are 1500mg as a single infusion, or as 1000mg on day 1 followed by 500mg on day 8, and is for the treatment of ABSSI 4. To find a suitable dosing regimen a Medline search was conducted using the keywords “dalbavancin,” and “prosthetic joint infection”. A phase 1 pharmacokinetic modelling study for osteomyelitis concluded that a two-dose, once-weekly regimen of 1500 mg IV dalbavancin on Day 1, and on Day 8 would provide sufficient distribution of drug into bone and articular tissue to have adequate tissue exposure over the dalbavancin minimum inhibitory concentration (MIC) for *Staphylococcus aureus* for 8 weeks 7. This dosing regimen was implemented in a phase 2 clinical trial in adult patients with osteomyelitis where 70 patients were randomized to dalbavancin, with safety and efficacy follow-up to 1 year. The 2-dose regimen of 1500 mg on day 1 and 8 was found to be well-tolerated and effective, with clinical cure of 97% at 6 weeks, and 96% at 6 months and 1 year 5.

As regards to safety, a clinical decision was made for the patient to receive both doses under medical supervision and with weekly blood test monitoring for 8 weeks. The long half-life of dalbavancin was a concern such that adverse events, if they do occur, could last longer, be more severe and possibly occur later than with a drug that has a shorter half-life. However, the phase 2 clinical trial of dalbavancin for osteomyelitis, where the same dosing was used (1500 mg on day 1 and day 8), the protocol only required blood tests at baseline, day 8, and day 28 with repeats as needed, and patients were followed out to 1 year, with no observed safety concerns 5. A Medline search for “dalbavancin” and “safety” identified a pooled analysis of phase 2 and 3 studies including 1778 healthy volunteers for the treatment of ABSSI 8. The lower dosing regimens for ABSSI were well tolerated, causing mild adverse events such as pyrexia, headache and nausea. Abnormalities in haematologic or biochemical parameters were similar to shorter acting comparators. The duration of adverse events was similar for dalbavancin and comparators, with a median of 3.0 and 4.0 days and a mean of 7.7 and 8.0 days, respectively. Additionally, the mean, median, and range of the time to the onset of adverse events were similar on each regimen. Therefore, dalbavancin-treated patients had similar duration and time to onset of adverse events as comparator-treated patients, despite the comparator antibiotics having much shorter half-lives (vancomycin, linezolid, cefazolin, nafcillin or oxacillin). There was no concern that our patient had an adverse event.

We conducted a brief cost analysis comparing inpatient vancomycin treatment and dalbavancin where the dose on day 1 is administered during an inpatient stay, and the second as a day attender at the hospital infusion suite, (Table 1). When considering the costs of medicines, hospitalisation, healthcare professional visits and monitoring, dalbavancin is a cost-effective option.

The main limitation is the uncontrolled nature of a case report and there remains little evidence to support a suitable dosing regimen for implant-related infections. The utilisation of debridement, prosthesis explantation, local antibiotics and 5 days of systemic vancomycin all will have contributed to a successful outcome. Until further evidence exists, we recommend dalbavancin should be considered as a reserve agent for infected EPR, to be used in cases of antimicrobial resistance where OPAT with alternatives such as daptomycin are unfeasible due to adverse reactions or intolerances.

Oral antimicrobials have recently been shown to provide a further option for outpatient treatment in selected cases where suitable oral regimens exist 9. In cases involving multi-drug resistance, dalbavancin may have a role in facilitating discharge and may be cost effective.

## Conclusion

In conclusion, we report a successful outcome when using dalbavancin for the treatment of an infected EPR. Drug costs were more than offset by avoiding the need for a prolonged inpatient stay and off-label dosing was well tolerated.

## Author contributions

Study design planning: TA SW.

Study conduct: TA SW.

Writing paper: TA.

Revising paper: SW TA AS JD.

### Ethical statement

Consent was received from the patient prior to submission for publication.

## Figures and Tables

**Table 1:** Cost analysis comparing inpatient vancomycin and dalbavancin treatment

	Dalbavancin	Inpatient Vancomycin (35 days)
Medicines cost* (excluding VAT)	3352.20	1207.50
Hospitalisation **	460.56	8050.00
Drug Assay costs ***	-	250.00
Total	3812.76	9507.50

*Drug costs are based on the British National Formulary accessed on 28^th^ June 2019. The cost of vancomycin is based on 1g administered every 12 hours for duration of treatment **Costs of hospitalisation is based on the National Health Service tariff *** Cost of vancomycin serum concentration measurement is based on £25 per sample and twice weekly monitoring.
